# Search for the optimized and key nephrometry elements combination in retroperitoneal laparoscopic partial nephrectomy: A retrospective study

**DOI:** 10.3389/fsurg.2023.1118971

**Published:** 2023-03-06

**Authors:** Yanyang Jin, Mingshuai Wang, Nianzeng Xing

**Affiliations:** ^1^ Department of Urology, Beijing Chaoyang Hospital, Capital Medical University, Beijing, China; ^2^ Department of Urology, The First Affiliated Hospital of Jinzhou Medical University, Jinzhou Medical University, Jinzhou, China; ^3^ Department of Urology, National Cancer Center/National Clinical Research Center for Cancer/Cancer Hospital, Chinese Academy of Medical Sciences and Peking Union Medical College, Beijing, China

**Keywords:** nephrometry, nomogram, exhaustive method, partial nephrectomy, scoring element

## Abstract

**Background:**

The nephrometry scoring system plays a key role in the preoperative evaluation of partial nephrectomy, and scoring systems based on anatomical characteristics have high similarity in scoring elements. Currently, there is little research on scoring systems related to retroperitoneal laparoscopic partial nephrectomy, and there is a lack of research on the combination of scoring elements, which requires further investigation.

**Methods:**

We retrospectively analyzed the clinical records of 107 patients who underwent retroperitoneal laparoscopic partial nephrectomy conducted by a single operator at a single center. The score and scoring elements were generated based on imaging. The scoring elements of each scoring system and all combinations of two to five elements were extracted. The predictive ability of different score combinations was evaluated by AUC value, and the key parameters of the score were found by taking the intersection. A nomogram was constructed and evaluated.

**Results:**

We observed that with an increase in scoring elements, the strongest combination of elements did not significantly increase the predictive ability of warm ischemia time (*P*>0.05), postoperative complications (*P*>0.05), and trifecta achievement (*P*>0.05). The combination of the maximum tumor diameter and the distance between tumor and collecting system or renal sinus had a good comprehensive predictive ability, and there is no significant difference with the traditional score (*P*>0.05). The nomogram generated according to this combination has an excellent prediction ability for predicting whether obtain trifecta of partial nephrectomy.

**Conclusions:**

Within the range of two to five elements, the critical degree of elements is more important than the number of elements. The maximum tumor diameter and the distance between the tumor and the collecting system or renal sinus was the key element of the prediction ability.

## 1. Introduction

Renal cancer is one of the most common urinary system tumors, and its incidence rate varies greatly across different regions worldwide. Europe, Australia, and North America have high incidence rates (1). Currently, although the indications for partial nephrectomy (PN) have been expanding (2, 3), its operation is relatively difficult, and complications cannot be ignored. The overall perioperative complication rate of PN is approximately 20%, according to previous reports (4, 5). Therefore, we need to conduct an adequate evaluation before PN and fully consider the wishes of the patient, the surgeon's experience and the hospital volume in the evaluation process. Nephrometry scoring (NS) systems can systematically evaluate and describe renal tumors based on their anatomical characteristics (6); the NS system was originally used to describe anatomical characteristics (6). In development, more and more centers and clinical studies have focused on its predictive ability of surgical results (7).

Presently, we lack a systematic comparison of the well-known NS systems for retroperitoneal laparoscopic partial nephrectomy (RLPN). Further, there is a lack of in-depth research on the scoring elements of renal tumors and the impact of the number of elements on the prediction ability of the scoring system, and whether the scoring elements have a more optimized combination, which are the key elements of the scoring system's prediction ability. In addition, no systematic research has been conducted in this area. Therefore, we retrospectively analyzed the demographic characteristics and perioperative results of patients who underwent RLPN performed at a single center by a single operator. Using an exhaustive method, we systematically studied the prediction ability of all combinations of the elements of these scoring systems. We then identified optimized combinations and key scoring elements to clarify the above issues and provide a reference and evidence for further optimization of clinical applications and scoring.

## 2. Materials and methods

### 2.1. Study population

We retrospectively reviewed the data of patients who underwent RLPN at Beijing Chaoyang Hospital between January 2014 and December 2017. All the patients had complete electronic medical records and imaging data and underwent abdominal enhanced computed tomography (CT) or enhanced magnetic resonance imaging with scanning thicknesses of 0.5 and 0.1 mm, respectively. Coronal, sagittal, and horizontal images were obtained to ensure the quality of the reconstructed images. In addition, arterial, venous, and delayed phase data were included to ensure accurate measurement of the elements in the score. Each patient was preoperatively diagnosed with a renal tumor by the imaging department. All patients or family members had no difficulty with communication and understanding and provided consent for the investigation and follow-up.

To ensure scoring accuracy, 40 patients who underwent enhanced CT at other institutions but did not have qualified electronic images were excluded. In addition, 3 patients with tumor metastasis and locally advanced disease who underwent palliative PN, 1 patient with polycystic kidney disease, 4 patients with severe pelvic and spinal deformities that affect surgery, 3 patients with a history of retroperitoneal surgery on the affected side, and others that severely affect surgical outcomes, and 1 patient with barriers to communication and comprehension and those who did not agree to be investigated and followed up were excluded.

### 2.2. Observe indicators

Demographic characteristics of the patients, including sex, age and Body Mass Index (BMI), were collected. The patient's enhanced CT or MRI is evaluated by an experienced urologist and the morphological features of the tumor are determined and evaluated according to established criteria. And then score is generated according to the observed characteristics of the tumor. The assessment includes RENAL (R.E.N.A.L) (6), PADUA (Preoperative Aspects and Dimensions Used for an Anatomical) (8), DAP (Diameter-Axial Polar Nephrometry) (9), NePhRO(zonal NePhRO scoring system) (10), SPARE(Simplified PADUA REnal) (SPARE) nephrometry (11), Mayo Adhesive Probability (MAP) (12) scoring and its scoring elements. Each patient's American Society of Anesthesiologists (ASA) score, Warm Ischemia Time (WIT), Operation time (OT), Estimated blood loose (EBL), and postoperative complications was obtained from raw surgical data and electronic medical records. Complications were graded according to the Clavien-Dindo (CD) criteria and the positive margin was determined by postoperative pathology. The criteria of trifecta of our center were taken as WIT less than 20 min, no postoperative complications greater than or equal to CDII, and negative resection margin (13). All procedures were performed by the same experienced urologist (Nianzeng Xing).

**Figure 1:**
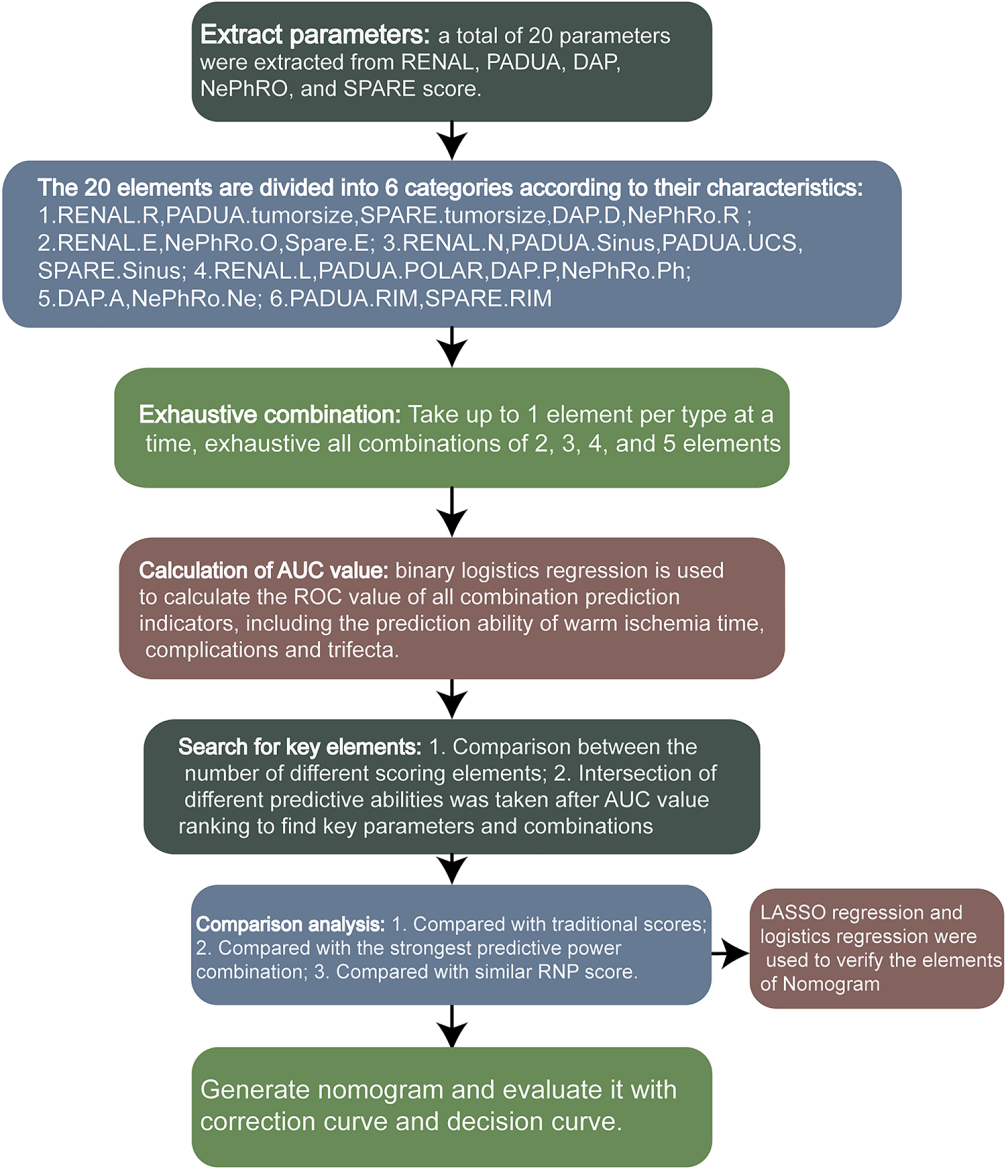
The analysis flowchart.

To summarize the elements of RENAL, PADUA, DAP, NePhRO and SPARE score, there are 20 elements in total, and some identical elements have been merged. We divided the 20 elements into 6 categories, including the elements related to the maximum tumor diameter and the elements related to the tumor exophytic rate. The relevant elements of the distance between the tumor and the collection system or the renal sinus, the longitudinal location, the depth of invasion of the tumor, and the parameters of renal rim, which are shown in Figure 1. Through R program, only one element is taken from each category at most each time. According to the number of element categories, it can be divided into 5 element combination, 4 element combination, 3 element combination and 2 element combination. All combinations of each combination type were listed by R procedure, and the total score was obtained to predict whether the WIT was longer than 20 min, whether the postoperative complications were greater than or equal to CD II, and whether the operation achieved trifecta. The Receiver Operating Characteristic (ROC) Area Under Curve (AUC) values were calculated to compare the predictive ability of all combinations. Through the analysis of the prediction ability, the key elements of the prediction ability are found. A nomogram was then constructed to predict whether a trifecta was achieved based on the key scoring parameters. Least absolute shrinkage and selection operator (LASSO) regression was used for regression analysis of clinical parameters and scoring elements to verify the elements involved in the construction of nomogram.

### 2.3. Statistical analysis

The continuous data of non-normal distribution were counted as median and Interquartile range (IQR). A univariate logistic regression model was used to evaluate the scores of each combination of scoring elements to predict whether the WIT was longer than 20 min, whether the postoperative complications were greater than or equal to CD II, and whether the operation achieved trifecta. ROC curve was generated. This process is automated by writing custom functions in R language and then generating summary tables to evaluate the predictive capabilities. Kruskall-wallis were used to test and analyze the overall difference distribution of AUC values between different parameter groups in predicting WIT, OT and Trifecta. Post hoc Dunn's multiple comparisons test was used for intra-group difference analysis. The calibration curve and decision curve are used to evaluate the nomogram. The difference of AUC value of ROC curve was tested by Z test. The AUC values and 95% Confidence interval (CI) of ROC curves were generated using pROC packages, The “rmda” package was used to generate decision curves, “rms” packages were used to generate calibration curves, and the “glmnet” packages were used for LASSO regression analysis. The statistical significance was set as *P* < 0.05. Venn diagram is obtained by uploading data to online website http://www.ehbio.com/test/venn/#/. SPSS 23.0 (SPSS Inc., Chicago, IL, USA), MedCalc 19.1 (MedCalc Software bv, Ostend, Belgium https://www.medcalc.org; 2019) and R4.1.2 [R Core Team (2021). R: A language and environment for statistical computing. R Foundation for Statistical Computing, Vienna, Austria. URL https://www.R-project.org/.] was used for statistical analysis.

## 3. Results

### 3.1. The process of comparing NS element combinations

A flowchart of the calculation process is shown in Figure 1. A total of 107 patients with complete perioperative clinical data and qualified imaging results were enrolled in this study and subsequently evaluated. All patients underwent RLPN followed by conventional renal artery clamping. In our cohort: 63(58.88%) patients had an intraoperative WIT of less than 20 min. Postoperatively, 91(85.05%) patients had no complication or complications lower than CD grade II and 105(98.13%) patients had negative margins. In total, 59(55.14%) patients obtained trifecta. Detailed demographic characteristics of the patients and the surgical results are shown in Table 1. The R program enumerated all the combinations of each combination type: 163 combinations of two elements, 692 combinations of three elements, 1,612 combinations of four elements, and 1,952 combinations of five elements.

**Table 1:** demography and perioperation feature of the 107 patients.

Variables	Value
No. patients	107
Age, years, median (IQR)	54.00(47–62)
Male gender, *n* (%)	70(65.42)
Female gender, *n* (%)	37(34.58)
Body mass index, kg/m^2^ median (IQR)	24.86(22.68–27.68)
Baseline eGFR, ml/min median (IQR)	88.14 (77.37–99.98)
Estimated blood loss, median, ml (IQR)	100(50–200)
Operation time, median, min (IQR)	80(60–105)
Warm ischemia time, mean, min (SD)	18.97(7.84)
Maximal tumor diameter, median, mm (IQR)	36.78(26.99–42.80)
Histological subtype, *n* (%):	
Clear cell	78 (72.90)
Papillary	4 (3.74)
Oncocytoma	1 (0.93)
Angiomyolipoma	14 (13.08)
Chromophobe	3 (2.80)
Benign cyst	4 (3.74)
Cystic renal cell carcinoma	2 (1.87)
Unclassified	1 (0.93)
Postoperative complications as Clavien–Dindo classification, *n* (%)	
Fever needs antibiotics (II)	4(3.74)
Hematuresis (II)	1(0.93)
Postoperative delirium (II)	1(0.93)
Hypoproteine (II)	2(1.87)
Blood transfusion (II)	3(2.80)
Urine leaks conservative treatment (II)	1(0.93)
Heart failure (II)	1(0.93)
Urine leaks need put stent (III)	1(0.93)
ICU (IV)	2(1.87)
Patients obtained Trifecta, *n* (%)	59(55.14)
Patients with WIT <20 min, *n* (%)	63 (58.88)
Patients with Complication ≥CD II, *n* (%)	16(14.95)
Patients with Negative margin, *n* (%)	105(98.13)

### 3.2. Overall comparison of the combined prediction ability of different element numbers

**Figure 2:**
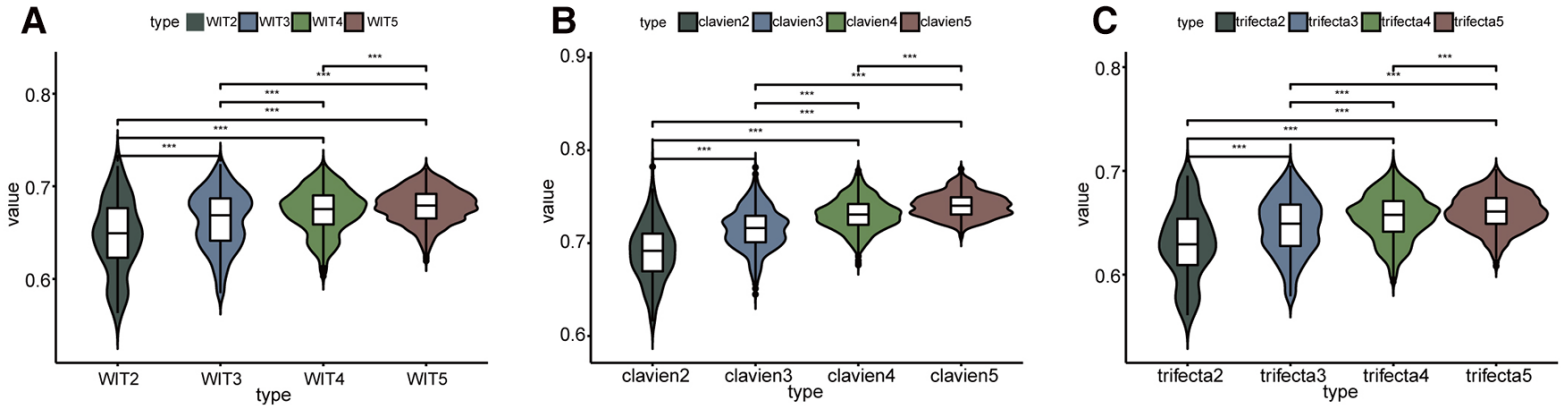
Comparison between the predictive ability of the number of different elements. WIT2 represents two elements combination to predict warm ischemia time of more than 20 min, WIT3 represents three elements combination to predict, and so on. clavien2 represents two elements combination to predict whether the postoperative complications were greater than or equal to CD II, clavien3 represents three elements combination to predict, and so on. *** Represents *P* < 0.001.

As shown in Figure 2 and Supplementary Table S1, the overall prediction ability increased with an increase in elements. Comparisons between groups were made using the Kruskall-Wallis test and Dunn's multiple comparison test. In terms of predicting whether the WIT was greater than 20 min, two-element combinations, three-element combinations, four-element combinations, and five-element combinations had significant differences in the whole and between groups (*P* < 0.001) (Figure 2A). In terms of predicting whether complications were greater than or equal to CD grade II after surgery, there were significant differences between the two-element combinations, three-element combinations, four-element combinations, and five-element combinations as a whole, and between groups (*P* < 0.001) (Figure 2B). In terms of predicting whether the operation achieved trifecta, two-element combinations, three-element combinations, four-element combinations, and five-element combinations had significant differences in the overall and inter-group comparisons (*P* < 0.001) (Figure 2C). Simultaneously, the AUC value of the strongest forecast combination showed no significant change with increased parameters in prediction of warm ischemia time (*P* > 0.05), postoperative complications (*P* > 0.05), and trifecta achievement (*P* > 0.05).

### 3.3. Identification of the key element combination according to the prediction ability

**Figure 3:**
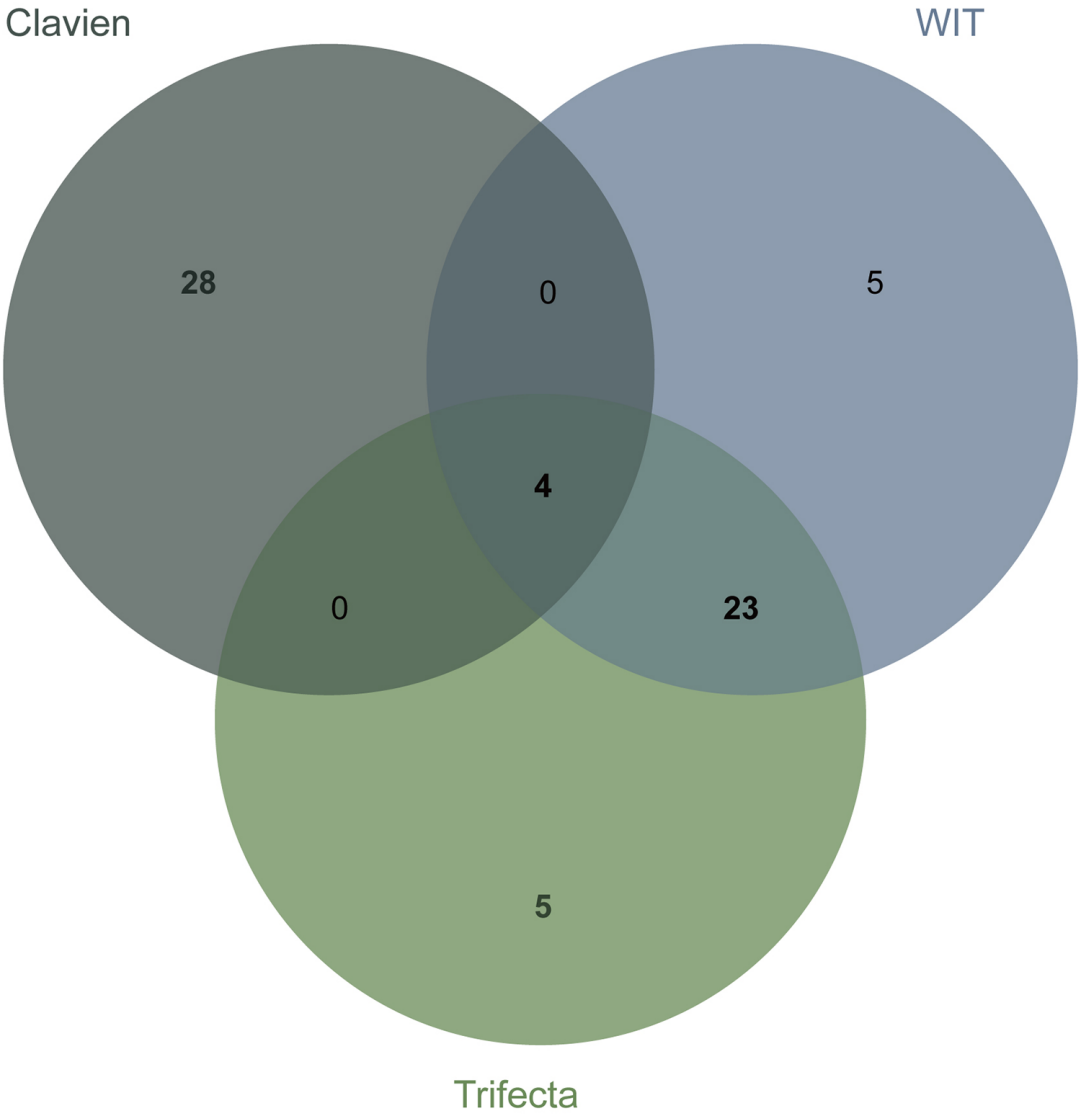
Identifying combinations of key elements by Venn diagram.

We observed that repeated intersection of the top element combinations predicted whether the postoperative complications were greater than or equal to CD grade II, whether the WIT was greater than 20 min, and whether the operation achieved trifecta. The optimal intersection prediction combination of three, four, and five elements has no obvious advantage over the existing score. By taking the intersection process, the two elements combination found and used to predict complications was greater than or equal to the CD grade II top 20% element combination and predicted whether warm ischemia time of more than 20 min in the top 20% element combination and whether the trifecta 20% element combination intersection can be obtained (Figure 3). The four combinations in the intersection were the DAP.D and RENAL.N combination, RENAL.R and RENAL.N combination, PADUA.tumorsize and RENAL.N combination, NePhRO.R and RENAL.N combination. Two elements in the intersection were a combination of the maximum tumor diameter and the distance between the tumor and the collection system or renal sinus. NePhRO.R and RENAL.N ranked first in predicting WIT in the two-element intersection combination, whereas DAP.D and RENAL.N ranked first in predicting complications in the two-elements intersection combination. NePhRO.R and DAP.D showed similar characteristics. Finally, the element combination of NePhRO.R and RENAL.N (hereinafter referred to as RN) was selected for the next comparison. Through the study of the influence of the number of elements on the predictive ability and the above screening process, RN was estimated to be the key combination of elements to predict complications, warm ischemia time, and whether the operation can achieve trifecta.

### 3.4. Comparative analysis of RN, various scoring systems and scoring element combinations

**Figure 4:**
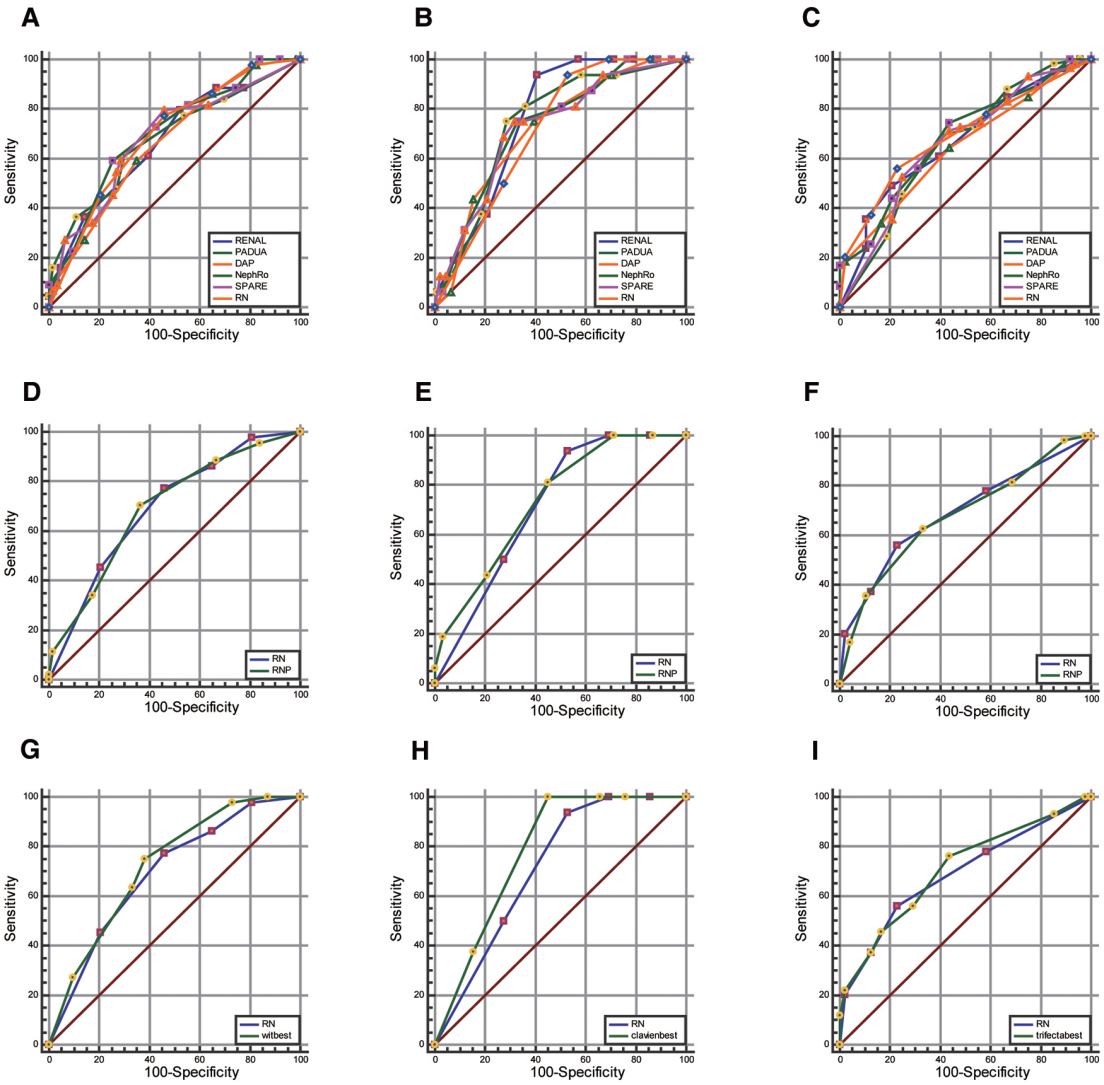
Comparison of AUC value between RN combination and traditional scores. (**A–C**), RNP score (**D–F**) and strongest element combination (**G–I**) in predicting warm ischemia time (wit >20 min, first column), postoperative complications (CD grade ≥II, the second column), and whether to achieve trifecta (Third column).

RN, RENAL, PADUA, DAP, NePhRO, SPARE, and RNP (R score, N score, and posterior perinephric fat thickness) scoring were compared. Regarding predicting whether the warm ischemia time was greater than 20 min, whether the postoperative complications were greater than or equal to CD II grade, and whether the operation achieved trifecta (Figures 4A–F and Supplementary Table S2). There were no significant differences in the AUC values of all the receiver operating characteristic curves (*P* > 0.05).

Through the exhaustive process, we observed that the combination of DAP.D + PADUA.UCS + NePhRo.Ne had the strongest predictive ability in predicting a prolonged WIT more than 20 min. RENAL.N + DAP had the strongest predictive ability in predicting whether there were postoperative complications greater than or equal to CD II grade. The combination of DAP.D + Spare.E + RENAL.N + NePhRo.Ne had the highest predictive value for determining whether the operation would obtain trifecta. The predictive ability of RN was slightly lower than that of the above combinations, and the difference was not statistically significant (*P* > 0.05) (Figures 4G–I and Supplementary Table S2).

### 3.5. A nomogram was established based on the maximum tumor diameter and the distance between the tumor and the collection system or the renal sinus to predict whether a trifecta could be obtained and evaluated

**Figure 5:**
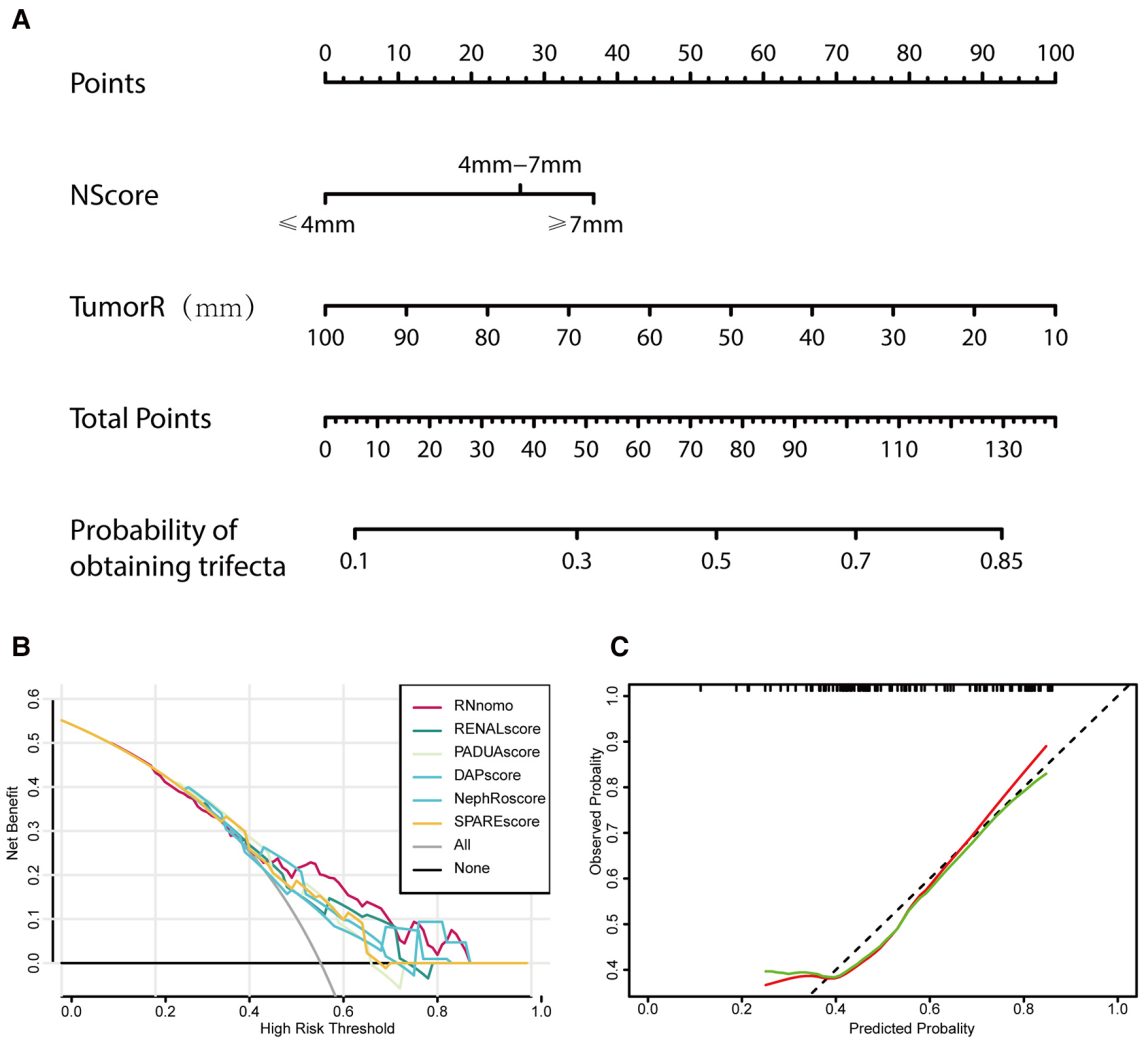
Tumor maximal diameter (NePhRO.R) and the distance between the tumor and the collecting system or renal sinus (RENAL.N) two elements are used to establish a nomogram (**A**), the decision curve is used to evaluate the predicted benefit by comparing the nomogram with the traditional scores (**B**), and the calibration curve is used to evaluate the deviation between the predicted value and the actual value of nomograms (**C**).

Based on these studies, a nomogram (Figure 5A) was established based on the binary logistic regression analysis to predict whether the operation could achieve trifecta, and the area under the receiver operating characteristic curve was calculated as follows: 0.708 (0.610–0.806). Compared with RENAL, PADUA, DAP, NePhRO, and SPARE scores in the decision curve, we observed that the nomogram constructed based on RN is slightly better than other scoring systems in terms of benefits (Figure 5B). However, by using calibration curve, we observed that the predicted RN value was close to the actual occurrence value, and the prediction ability of the low trifecta rate was relatively poor (Figure 5C).

### 3.6. LASSO regression, univariate and multivariate regression analysis were used to analyze and verify the scoring elements

**Figure 6:**
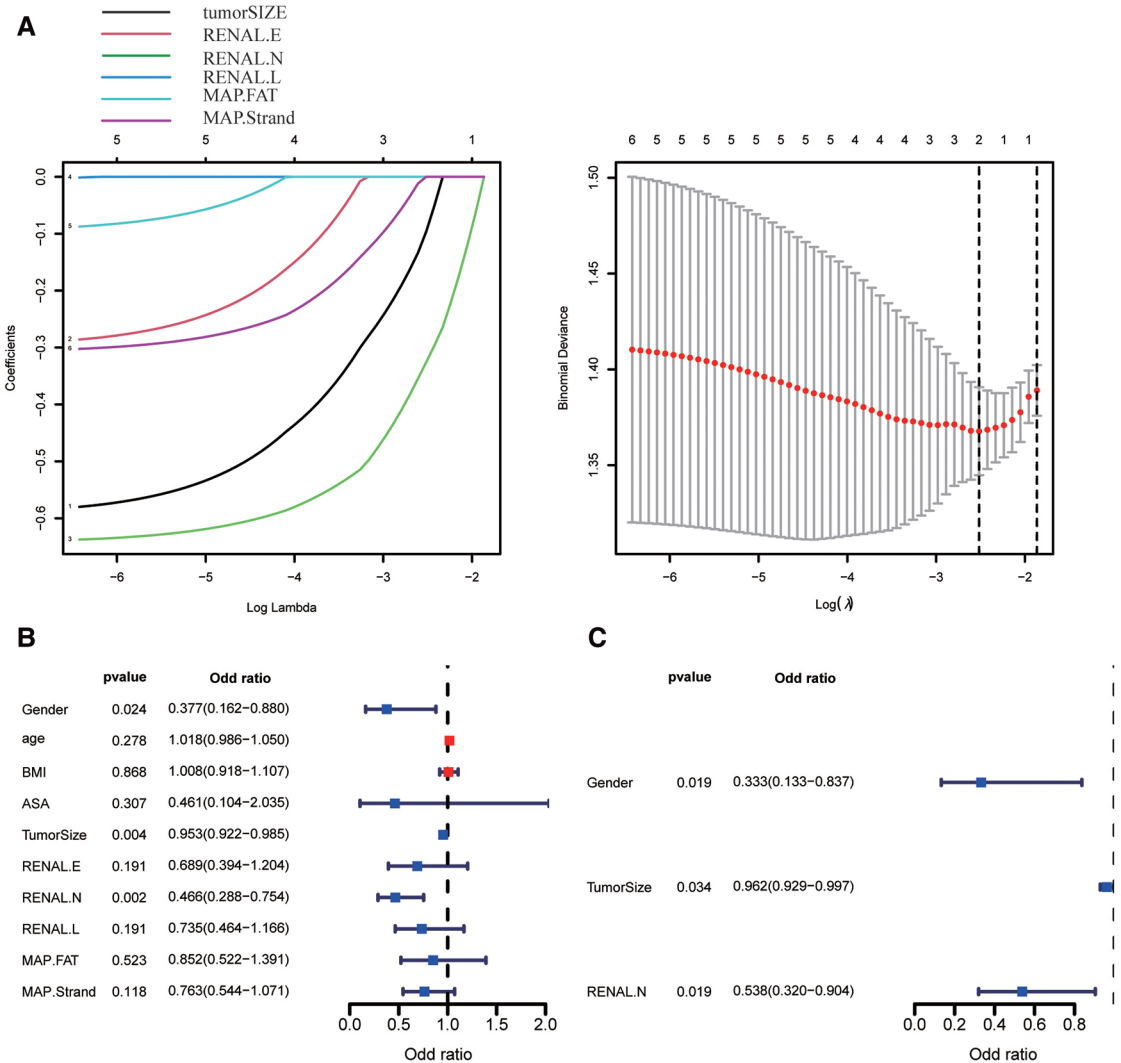
Screening the key factors of clinical variables and scoring elements in predicting whether to obtain trifecta by using lasso regression (**A**), univariate (**B**) and multivariate regression analysis (**C**). (In Figure 6A, 1–6 are tumorSIZE, RENAL.E, RENAL.N, RENAL.L, MAP.FAT, and MAP.Strand, respectively).

According to our studies, RN is considered to have a strong predictive ability for trifecta. LASSO regression and multivariate regression analyses were used for further verification. In consideration of collinearity, maximum tumor diameter (tumorSIZE), RENAL.E, RENAL.N, RENAL.L, and MAP. Fat (posterior perinephricartery fat thickness score element in the MAP score) and MAP. strands (MAP Stranding score element) were included in the LASSO regression analysis. The maximum tumor diameter and RENAL.N were ultimately included in the model by LASSO regression analysis (Figure 6A). After LASSO regression, univariate and multivariate logistic regression analyses were conducted for sex, age, body mass index, ASA(American Society of Anesthesiologists) score, maximum tumor diameter, RENAL.E, RENAL.N, RENAL.L, MAP.FAT, and MAP.Strand. In the univariate analysis, sex, maximum tumor diameter, and RENAL.N were risk factors for predicting trifecta in PN (Figure 6B). The odds ratio (OR) and 95% CI values were 0.377(0.162–0.880, *P* = 0.024), 0.953(0.922–0.985, *P* = 0.004), and 0.466(0.287–0.754, *P* = 0.002), respectively. In the multivariate analysis, sex, maximum tumor diameter, and RENAL.N were independent risk factors for predicting PN trifecta (Figure 6C). The OR and 95% CI values were 0.333 (0.133–0.837, *P* = 0.019), 0.962 (0.929–0.997, *P* = 0.034), and 0.538 (0.320–0.994, *P* = 0.019), respectively.

## 4. Discussion

The NS system has important guiding significance in preoperative evaluation, patient consultation, and the selection of surgical methods for renal cancer (7). There are more than 20 well-known scoring systems available. Most NS systems are generated based on the anatomical characteristics of renal tumors (14). Through comparison, we observed that these anatomical factors were similar. The differences between different scoring systems are mainly in the selection of scoring elements, different score assignments of the same or similar elements, and different definitions of anatomical elements. The NS system is not entirely a mechanical formula or a mathematical tool. One of the important functions of the scoring system is to standardize and comprehensively describe the key anatomical characteristics of tumors from all aspects (6, 15), which can facilitate communication and comparative analysis. The NS system is also a predictor of long-term effect (16). Research on the predictive ability of the NS system has not weakened since the development of the scoring system (17, 18). To improve the predictive ability of the scoring system, the new scoring system has been tried with many methods, including adding parameters (18), simplifying parameters, and modifying scoring through regression analysis (11).

RLPN has characteristics that are distinct from those of transperitoneal laparoscopic partial nephrectomies (TLPN). The space in the posterior peritoneum is relatively narrow, and although it is more difficult to resect a tumor in the lower pole than in the upper pole, RLPN facilitates the exposure of the renal artery without occlusion of the renal vein. Thus, RLPN is a good surgical approach because of its characteristics and advantages. If the surgeon has sufficient experience and the proper technique is applied, compared to TLPN, the operation time and blood loss of RLPN may be shorter or less, and the postoperative results and oncologic effects of these techniques are similar (19, 20). Moreover, despite advances in robot-assisted surgery, RLPN is still the standard and most popular procedure in many areas (21).

After more than 10 years of development, the parameters that currently exist in the scoring system have proven their value through repeated studies. However, systematic research on these parameters is lacking. In this study, we observed that when the number of elements was between two and five, the more elements participating in the construction of the scoring system, the stronger the overall prediction ability and the more concentrated the distribution of the predicted AUC value. However, by comparing the combination of the strongest prediction ability of different scoring combinations, we observed that within the range of two to five elements, the critical degree of elements is more important than the number of elements. Based on the above findings, the increase in elements may more comprehensively reflect the anatomical characteristics of renal tumors, whereas excessive scoring elements may interfere with the weight and predictive ability of key elements. Thus, based on the above speculation, there may be key elements or combinations of key elements that affect prediction ability. Furthermore, through the intersection of the combinations of two elements with the highest predictive ability, we observed that the maximum tumor diameter and the distance between the tumor and the renal sinus or collecting system might be the key elements of predictive ability.

To test this hypothesis further, we compared several aspects. In comparison, we observed that the predictive ability of RN was slightly lower or better than that of the traditional scoring system with the least elements, and there was no significant statistical difference. RN's comprehensive prediction ability is strong, and we believe it is the key element of the prediction ability of the scoring system. Based on this assumption, we constructed a nomogram to predict whether trifecta could be achieved through binary logistic regression analysis. By comparing the decision curve with other scoring systems, we discovered that the nomogram constructed based on RN was similar to the other scores and had a strong ability to predict a higher rate of trifecta in surgery. However, the relative ability to predict a low trifecta rate during surgery was poor. Furthermore, although the nomogram constructed by RN was slightly better than the other scoring systems in terms of clinical benefits, the difference was small.

In addition, because the algorithm used in this study was not conventional, we used LASSO regression analysis to screen and verified that the maximum tumor diameter and the distance between the tumor and the collecting system or the renal sinus were appropriate parameters for the construction of the prediction model. Also, we performed univariate and multivariate regression analyses and discovered that sex, maximum tumor diameter, and RENAL.N were independent risk factors for predicting whether trifecta was achieved, and the validation of the various algorithms further confirmed the validity of the exhaustive method.

The results obtained from the exhaustive methods are supported by previous studies. In a study by Bai et al. (22), large maximum tumor diameter and PADUA score of medium and high complexity were correlated with lower PN trifecta rates. However, in a multicenter study of 147 completely endogenous renal tumors undergoing partial nephrectomy, it was discovered (23) that the maximum tumor diameter was the only risk factor for predicting trifecta (OR: 0.667, 95% CI: 0.66–0.79, *P* < 0.001). However, a retrospective study of 482 patients who underwent partial nephrectomy conducted by Bianchi et al. demonstrated (24) that the ASA score, whether it involved the collection system and the surgical method were independent risk factors for achieving trifecta. In another retrospective study of 68 patients who underwent laparoscopic partial nephrectomy (25), tumor size and the surgeon's learning curve were prognostic factors for achieving trifecta. In a retrospective study of 147 patients who underwent partial nephrectomy, Sciorio et al. found (26) that the maximum tumor diameter could predict trifecta, warm ischemia time, and blood loss. Gu et al. (27) discovered that in patients undergoing laparoscopic partial nephrectomy, maximum tumor diameter, distance from the collection system or the renal sinus, and preoperative glomerular filtration rate were independent prognostic factors for achieving pentafecta. Huijiang Zhang et al. (28) conducted an enumeration-based clinical study on the elements involved in the previous PN-NS system. Three scoring systems have been developed to predict surgical options (radical or partial nephrectomy). Invasion of the collecting systems and renal sinus was also a key predictor of the surgery type. This method provides a good idea for the construction of clinical models with few variables and relatively less computation. However, in a multicenter clinical study of robot-assisted partial kidney resection by Bhandari et al. (29), the model was constructed using logistic regression, random forest, and neural networks. For predicting intraoperative events, the optimal model had an AUC value of 0.858, using 1,690 patients and 38 variables. The best model predicted postoperative events with 59 variables for 1,406 patients and an AUC value of 0.875. The accuracy of these models was high. Owing to their greater predictive power, these models may be gradually applied in clinical settings in the future.

The ability of maximum tumor diameter to predict warm ischemia time and trifecta has been widely recognized in clinical practice, and some studies even believe that the predictive ability of maximum tumor diameter is better than that of other scoring systems (30, 31). The relationship between the tumor and the renal sinus or the collection system reflects the depth of tumor invasion. Once the collection system or the renal sinus is invaded, the difficulty of inserting the intraoperative suture will increase, and the incidence rate of postoperative bleeding, infection, urinary leakage, and other complications may also increase (24). Tumor exophytic properties and longitudinal tumor position were widely included in the score. Presently, with the aid of endocavitary ultrasound (32), skilled surgeons can achieve good results in the treatment of completely endogenous tumors (33). The position of the longitudinal axis of the tumor has also been studied, and it is often difficult to deal with the upper renal pole using the transperitoneal approach (10). However, in the retroperitoneal approach, the lower pole of the renal tumor is often difficult to manage, and because the space is relatively small, the suture angle is too difficult to operate on. Nonetheless, it can be handled better with the help of renal “Polar Flip” or “Renal Pedicle Rotation” technology (34, 35). The MAP score or the scoring elements of posterior perinephric fat thickness and stranding type in MAP score are often included in the new score or nomogram (36, 37). In our study, we also tried to add the posterior perinephric fat thickness score, and its predictive ability was not significantly improved after addition. In a study by Kawamura et al. (38), the MAP score was associated with the presence of adhesive fat and intraoperative blood loss in an Asian population but not with postoperative complications.

This study has some limitations. First, it was a single-center study, and the number of cases operated by a single surgeon is limited. Moreover, all patients in our cohort we employed the on clamp technique, however, appropriate use of the off clamp technique resulted in better preservation of renal function (39). On the other hand, we lacked dataset validation from other centers. Further, our main evaluation index was the AUC value, which, although relatively simple, may be biased and cannot fully reflect the prediction ability of the score. In addition, the clinical data we collected were not sufficiently comprehensive. For example, the preoperative, intraoperative, and postoperative conditions of patients' renal function should be comprehensively considered, but these aspects are not completely designed. Furthermore, we lack long-term follow-up assessments after surgery, such as the ROMe's achievement (16). Finally, despite the fact that a larger number of isotype operation have been performed by the surgeon before data collection, continuous progress is being made and the learning curve of the surgeon can have an impact on the surgical outcome.

In conclusion, as far as we know, this is the first study to systematically describe the influence of the number of elements involved in the construction of a PN-NS system on predictive power. Moreover, this is the only clinical study in which the maximum tumor diameter and the distance between the tumor and the renal sinus or the collecting system are key factors in predicting trifecta, complications greater than or equal to the CD grade II, and prolonged ischemia time, based on the exhaustive methods. Our research method is highly innovative, and the key elements we discovered and established are conducive to the preoperative evaluation, consultation, and selection of surgical methods. Because of the limited number of patients enrolled, the applicability and reliability of our model need further investigation. Our model requires much larger statistical calculations of clinical data and data validation from other centers. In the future, large amounts of data could be further studied and analyzed using machine learning. Consequently, clinical experience and data in partial nephrectomy will be rationally utilized, and the scoring system will be continuously improved and optimized through various algorithm analyses. We believe the model will provide more effective information for clinical use, help in clinical decision-making, and complete more high-quality operations to maximize patient benefit.

## Data Availability

The original contributions presented in the study are included in the article/**Supplementary Material**, further inquiries can be directed to the corresponding author/s.
