# Supplementary material for: Search for the optimized and key nephrometry elements combination in retroperitoneal laparoscopic partial nephrectomy: A retrospective study
Source: Front Surg. 2023 Mar 6;10:1118971. doi: 10.3389/fsurg.2023.1118971 (PMC10025499; doi:10.3389/fsurg.2023.1118971)
Supplement: Supplementary file 1 [file Table1.docx]

Supplementary table 1, profiles of AUC values for different parameter combinations (WIT2 represents 2 elements predicting warm ischemia longer than 20min, WIT3 represents 3 elements predicting warm ischemia longer than 20min, and so on).

| Prediction parameters | AUC values median | AUC values IQR (25%-75%) | AUC Highest value |
| --- | --- | --- | --- |
| WIT2 | 0.649 | 0.623-0.676 | 0.722 |
| WIT3 | 0.669 | 0.641-0.687 | 0.724 |
| WIT4 | 0.676 | 0.658-0.690 | 0.720 |
| WIT5 | 0.679 | 0.665-0.692 | 0.720 |
| Clavien2 | 0.692 | 0.670-0.710 | 0.782 |
| Clavien3 | 0.716 | 0.701-0.729 | 0.782 |
| Calvien4 | 0.731 | 0.720-0.742 | 0.778 |
| Calvien5 | 0.740 | 0.731-0.749 | 0.780 |
| Trifecta2 | 0.629 | 0.609- 0.654 | 0.695 |
| Trifecta3 | 0.649 | 0.628- 0.668 | 0.705 |
| Trifecta4 | 0.658 | 0.6418- 0.671 | 0.706 |
| Trifecta5 | 0.661 | 0.649- 0.674 | 0.702 |
