# Supplementary material for: Search for the optimized and key nephrometry elements combination in retroperitoneal laparoscopic partial nephrectomy: A retrospective study
Source: Front Surg. 2023 Mar 6;10:1118971. doi: 10.3389/fsurg.2023.1118971 (PMC10025499; doi:10.3389/fsurg.2023.1118971)
Supplement: Supplementary file 2 [file Table2.docx]

| NS systems or combination of scoring elements | WIT＞20min（AUC 95%CI） | Complication≥Clavien dindoⅡ  （AUC 95%CI） | Obtain Trifecta  （AUC 95%CI） |
| --- | --- | --- | --- |
| NePhRO.R+RENAL.N | 0.693（0.596-0.779） | 0.718(0.623-0.800) | 0.689（0.592-0.775） |
| RENAL | 0.672（0.574-0.759） | 0.764（0.672-0.840） | 0.669（0.571-0.757） |
| PADUA | 0.681（0.584-0.768） | 0.744（0.650-0.823） | 0.656（0.558-0.745） |
| DAP | 0.665（0.567-0.754） | 0.719（0.624-0.801） | 0.641（0.543-0.732） |
| NePhRO | 0.704（0.608-0.789） | 0.732（0.638-0.813） | 0.682（0.585-0.769） |
| SPARE | 0.684（0.587-0.770） | 0.726（0.631-0.808） | 0.659（0.561-0.748） |
| RNP | 0.692（0.596-0.778） | 0.735（0.641-0.816） | 0.678（0.581-0.765） |
| DAP.D+PADUA.UCS+NePhRo.Ne | 0.724（0.629-0.806） | - | - |
| RENAL.N+DAP.P | - | 0.782（0.692-0.856） | - |
| DAP.D+Spare.E+RENAL.N+NePhRo.Ne | - | - | 0.706（0.611-0.790） |

Supplementary table 2 Comparison of AUC value between RN combination and traditional scores（A-C）, RNP score （D-F）and strongest element combination （G-I）in predicting warm ischemia time(wit > 20min, first column), postoperative complications(Clavien Dindo ≥ II, the second column) and whether to achieve trifecta(Third column).
